# Assessing a child or adolescent with low back pain is different to assessing an adult with low back pain

**DOI:** 10.1111/jpc.15933

**Published:** 2022-02-26

**Authors:** Joshua W Pate, Rhiannon Joslin, Karen Hurtubise, David B Anderson

**Affiliations:** ^1^ Graduate School of Health University of Technology Sydney Sydney New South Wales Australia; ^2^ Faculty of Health Sciences University of Southampton Southampton United Kingdom; ^3^ CanChild Centre for Childhood Disability Research, School of Rehabilitation Sciences McMaster University Hamilton Ontario Canada; ^4^ Faculty of Medicine and Health, Sydney School of Health Sciences University of Sydney Sydney New South Wales Australia

## Abstract

In contrast to an assessment of an adult presenting with low back pain (LBP), clinicians should utilise different approaches when assessing children and adolescents presenting with LBP. Children are not ‘little adults’. There are some unique pathologies that only occur in this age group: (i) *serious pathologies* include infection, fracture, child abuse and malignancy; (ii) *growth‐related pathologies* include scoliosis, Scheuermann's disease, pars fracture and spondylolysis; and (iii) *rheumatological conditions* include juvenile idiopathic arthritis and ankylosing spondylitis. With changes in each child occurring physically, emotionally and socially, a clinician's knowledge of typical developmental milestones is essential to identify regression or delayed development. When listening to a child discuss their pain experience, a flexible structure should be implemented that gives the capacity to actively listen to a child's narrative (and that of their guardian) and to conduct an effective physical examination. This viewpoint also summarises the relationship between potential clinical diagnoses and key elements of a physical examination. Deciding on the type and timing of paediatric‐specific physical examination tests requires unique child‐centred considerations. Paediatric‐specific outcome measures should be used but implemented pragmatically, with consideration regarding the time, complexity and pathology suspected. Systematic and rigorous approaches to both treatment planning and re‐assessment are then proposed for the assessment of children and adolescents presenting with LBP.

## Introduction

In terms of spinal pathologies, children should not be assessed as ‘little adults’. Beyond pathologies that do relate to both children and adults, such as disc pathology, herniated discs and lumbar radiculopathy, there are a number of unique pathologies that only arise in childhood. Detecting the unique spinal pathologies that exist in children requires clinicians to be aware of how these conditions present and to adapt their clinical assessment to the child's situation.

*Serious pathologies* require careful history taking and examination to identify any red flags. These can include infection, fracture, child abuse and malignancy. *Growth‐related pathologies* relate to the physical changes that occur within a child's maturing musculoskeletal system through growth and puberty, making childhood a susceptible period for injury. Examples include scoliosis, Scheuermann's disease and spondylolysis ± spondylolisthesis.^1^ *Rheumatological conditions* are another category of pathologies that must be considered in the assessment and treatment of a child, such as juvenile idiopathic arthritis (JIA) and ankylosing spondylitis.

Only 5–15% of low back pain (LBP) in adults has a known cause of pathology (serious or specific), whereas the rate of known causes of pathology in children and adolescents varies dramatically in recent literature; from an upper range of 52–73% of all LBP^2^ to a lower range of 1–5%.^3^, ^4^ Adult literature has established the existence of spinal pathology on magnetic resonance imaging in asymptomatic populations that increases with age.^5^ While fewer studies have been completed in paediatric populations, they have suggested that spinal pathology can exist without symptoms.^6^ Further, disc pathology appears to fit a pattern that increases with age, with a prevalence of 22% in those under 18 years.^7^ Contrary to disc pathology, pars fracture/spondylosis were more likely to be associated with symptoms with lower prevalence in asymptomatic populations.^7^

Although children and adolescents may have higher rates of specific pathology, they potentially^8^ report LBP at a lower prevalence to adults, with a prevalence rate of 20–47%,^1^, ^9^ compared with 80% in adults. Similarly, the prevalence of consultations for back pain increases with age throughout childhood and into adulthood.^10^ With changes in each child occurring physically, emotionally and socially, a clinician's knowledge of typical developmental milestones is essential to identify regression, delayed development or functional changes. For example, one study showed that malignant spinal cord compression peaked in those under 3‐years, motor deficit occurred in all cases and would have resulted in regression of motor milestones; however, only 59% of cases reported pain.^11^ Paediatric pain experiences are uniquely impacted by the dynamic interplay between biological (e.g. puberty, gross motor skill milestones and growth), psychological (e.g. cognitive and affective milestones) and social (e.g. friends, sense of identity, school and family) factors, throughout different childhood ages. Therefore, LBP has a wide range of clinical presentations in children and adolescents. For example, serious underlying pathologies are more common pre‐puberty, and non‐specific LBP (with no demonstrable pathological cause) is more likely in adolescence.^12^ A thorough understanding of the incidence, prevalence and prognosis of pain‐related conditions throughout childhood can helpfully inform clinical reasoning.

A comprehensive and systematic assessment of children and adolescents with LBP is important in progressively refining a diagnostic hypothesis, as well as informing prognosis and treatment strategies. It involves listening to a child's narrative through a child‐centred lens^13^ and conducting a structured physical examination.^14^ This viewpoint serves to summarise the key features of a clinical assessment of children and adolescents (aged 0–19) presenting with LBP.

### Subjective examination

Children and adolescents are commonly brought to appointments by their guardian, and while their perspective is helpful and even essential for younger ages, it is vital to establish the child's voice. This might be the first time a young person has encountered a health‐care professional, and unlike adults, children are unlikely to have an expectation of what is required of them or how best to communicate their needs and wishes. Conducting an assessment on a child with LBP therefore requires a unique set of approaches that are relevant to differences in how pain manifests in children and adolescents. Questioning about a child's experience of pain during the assessment should cover a broad range of topics, such as those outlined in Table 1. Commencing the interview with a broad open‐ended question like ‘*Tell me about why you have come to see me today?*’ allows the child to tell ‘their’ story. Attentive active listening is a skill that improves validation of a child and their experience. Attempts at triangulation of key ideas, *via* strategies such as drawing tasks (e.g. ‘*Draw whatever you think of when you hear the word “pain”*’, or labelling a body chart diagram^15^) or non‐verbally showing areas of pain with their hands may also facilitate an empathetic interaction which can improve therapeutic alliance and treatment compliance.

**Table 1:** Common elements of listening to a child's narrative about their experience of pain and clinical reasoning

Element	Key purposes	Rationale
Onset of symptoms		
‘When/How did it happen?’ ‘Did anything else change in your life during that time?’	Identify sudden versus gradual onset Identify involvement of trauma, the mechanism of the trauma and management to date	Differentiation between traumatic and non‐traumatic pathology Traumatic injuries could include fracture, herniated disk, slipped apophysis, spondylolysis ± spondylolisthesis or muscle strain Gradual onset may be more indicative of scoliosis or Scheuermann's disease
	Identify a recent increase in physical load, for example puberty/growth spurt, addition of new sport, return to sport from break, increase in training	Recent growth period can be associated with musculoskeletal changes but also growth‐related conditions such as scoliosis or Scheuermanns AS and spondylosis can be associated with an increased training load
	Identify a recent psychological or social load placed on young person, for example recent parental divorce, transition to new school or friendship/relationship difficulties	Planning future management
	Identify recent infection or illness	Consider reactive arthritis, infection, osteomyelitis
	Identify any incongruence with history (red flag)	Follow safeguarding procedures as required
Pain characteristics		
Location of pain, description and aggravating and easing factors	Identify anatomical structures associated with pain and symptoms	Spondylosis normally specific lower back pain associated with sport and/or activity Burning or shooting radiating pain into buttock or leg can suggest radicular pain from herniated disk, slipped apophysis, spondylolysis ± spondylolisthesis
24‐h pattern associated joint swelling Other pain locations Associated sore eyes (also see below red flags)	Identify pathophysiology	JIA and AS associated with early morning stiffness, may have joint swelling and with juvenile AS, enthesitis is more common and presents similarly to Severs disease or Osgood Schlatters. Uveitis can present with JIA
Red flag questions		
Pain characteristics Constant unremitting pain Night pain, pain affecting sleep (waking and/or interrupting) Associated with headaches ± nausea/vomiting Change in child's behaviour – irritable, quiet, poor sleep or increased sleep/fatigue Regression in motor milestones (loss of a skill such as handwriting or speech and language) Systemic symptoms Fever weight loss, reduced appetite, sweating, other system involvement (respiratory, incontinence, etc)	Identify serious pathology Malignancy, tumours Systemic illness infection Some forms of JIA and multisystem inflammatory diseases such as vasculitis Neurological disease/event	Requires ongoing medical investigation
Treatment to date		
Investigations Diagnoses Expectation and beliefs of young person and family relating to the diagnosis, management and concept of pain	Confirm and validate the young person's treatment journey so far Identify further medical intervention if required Identify level of understanding of child and guardian regarding their current health and ongoing management	Planning possible future investigations and treatments Enabling targeted pain science education
Impact of pain/symptoms		
Ability to perform meaningful activities like hobbies and sports School attendance and school functioning Friendships and spending time with friends Effect on mood and general wellbeing. Changes in ‘self’	Identify the biopsychosocial impact of the pain to the person Understand what factors are important to the young person Identify contributing factors to the pain experience including victimisation, anxiety, low mood or stress (e.g. issues with teachers and/or friends)	Identifying possible shorter‐term therapy goals Considering referral to clinical psychologist Considering involvement of school personnel
Family history		
Of musculoskeletal conditions/chronic pain	Understand social influences and congenital anomalies	Planning future management
Of rheumatoid arthritis/AS	Identify pathophysiology	Raises suspicion of JIA and AS
Past medical history		
Birth history including prematurity and time spent in high dependency/special care	Identify possible birth trauma or pathology that could be linked, for example neurological involvement or spina bifida	Potential for ongoing medical investigation
Developmental history e.g. did the young person meet normal developmental milestones physically, socially and cognitively	Identify delayed development and/or special educational need	Adapt physical examination to needs of child
Previous level of functioning		
The number of sports, at what level and how often a child participates per week School attendance Hobbies and other meaningful activities (e.g. part‐time job in adolescents)	Identify increased risk of sport‐related spinal pathology	Spondylosis more prevalent in young athletes who play multiple sports but especially sports involving spinal extension Identifying possible longer‐term therapy goals such as school, sport, hobbies, work

AS, ankylosing spondylitis; JIA, juvenile idiopathic arthritis.

Other strategies include creative questions, such as ‘*If you were to assign a colour to your pain, what would it be?*’, or ‘*If you had to picture your pain as an animal what would it be? Why?*’ Further, to target a self‐management approach it has been suggested to ask ‘*What do you do to help your pain?*’ followed by ‘*What can we do to help your pain?*’.^16^ Health‐care professionals also have a duty of care to keep children safe and need to be aware of common signs of non‐accidental injury, such as incongruence of history, and escalate any safeguarding concerns appropriately.

Other considerations during an assessment, which are consistent with child‐centred approaches relate to fostering an environment where a child is comfortable providing information. Clinicians may consider encouraging one or more guardian(s) be present during the consult, to increase a child's comfort levels in sharing information about their experience, and to ask questions and provide input, because guardians also offer a unique perspective of their child. However, this approach can result in interruptions or increase pressure on children and adolescents. Multidisciplinary pain clinic assessments often balance these two issues by including a moment where a child leaves the consultation room as part of a functional assessment (e.g. walking in the hallway), thereby providing an opportunity for the clinician to allow a child to discuss information they may not feel comfortable providing in their guardian's presence. During this time, the guardian in the consultation room can then provide further subjective information and clarifications. At the conclusion of the history section of the initial assessment, a warning should be provided to children and adolescents that symptoms may be exacerbated by the physical examination.

### Physical examination

A thorough physical examination provides important support for or against a suspected pathology. Previous research has described a range of different elements of a physical examination in children to identify red flags, assist with diagnosis and help to plan treatments.^17^ Common elements of a systematic physical examination are: (i) observation (static and dynamic); (ii) active range of motion; (iii) passive range of motion; (iv) isometric muscle testing; (v) manual muscle testing; (vi) passive accessory movement testing also called ‘Joint Play’; (vii) neurological screening; (viii) special tests; (ix) functional tests; and (x) palpation. Observational and performance‐based outcomes have also been promoted in recognition of discrepancies between self‐report and performance in paediatric pain populations.^18^ Table 2 summarises the relationship between potential clinical diagnoses and key elements of a physical examination.

**Table 2:** The relationship between potential clinical diagnoses and key elements of a physical examination

Potential clinical diagnosis	Key elements of physical examination
Neurological involvement, for example radicular pain, potential spine or brain tumour	Neurological Examination – motor, sensory and reflex testing including abdominal reflexes. Upper motor neurone testing – Babinski and conus Observation of play/functional tasks like walking and getting off the floor to identify motor and sensory dysfunction Observation of behaviour to indicate pain or discomfort. Neurosensitivity tests, for example straight leg raise
Rheumatological disease, for example JIA and AS	Complete pGALS of all joints Palpation to identify specific tenderness, heat and/or swelling of joints or enthesitis Observation – check for rashes and changes to nails
Systemically unwell, for example malignancy, infection	Complete pGALS as a screen for rheumatological disease Observation of play/functional tasks like walking and getting off the floor to identify motor and sensory dysfunction or difficulty Observation of behaviour to indicate pain or discomfort
Scoliosis or Scheuermann's	Observation in standing to identify curve/kyphosis Scoliosis more prominent in forward flexion, from behind the person asymmetry can be observed more clearly Laying prone the kyphosis associated with Scheuermann's can be more apparent In prone the spine can be palpated to identify a curve associated with a scoliosis ± associated pain
Muscular strain	Expect pain with active movements but not/or less with passive movements. Pain may be associated with a particular movement (specific muscle or group) that is made worse if resistance to this movement is increased
Spondylosis	Pain on palpation over specific area of lumbar vertebrae ± muscle spasm guarding Pain with active lumbar extension and/or rotation Observation of increased lumbar lordosis

AS, ankylosing spondylitis; JIA, juvenile idiopathic arthritis; pGALS, paediatric Gait Arms Legs and Spine.

It is important to understand and physically assess for typical findings of specific conditions. For example, in JIA, cervical involvement is a serious and persistent manifestation of JIA with severe sequelae, yet children rarely complain of pain. The paediatric Gait Arms Legs and Spine is a brief and evidence‐based assessment to detect abnormal joints in school‐aged children and young people.^19^

### Imaging and clinical tests for LBP

In general, children and adolescents (like adults) are not recommended to be sent for imaging or laboratory studies in the absence of suspected serious pathology.^20^ Decreasing the use of imaging in children and adolescents is difficult, however, given the higher percentage of specific and serious pathology among those presenting with LBP.^12^ Therefore, radiation‐saving measures such as magnetic resonance imaging and lower dose radiation X‐ray imaging (such as EOS) are prudently used in such scenarios. Laboratory studies (i.e. blood tests) and specialist referrals may be indicated in the presence of signs of possible inflammatory pathologies, such as morning stiffness, rigid kyphosis and tenderness over the sacroiliac joint, or malignancy.

### Outcome measures

Different outcome measures are also required when assessing LBP in children and adolescents. Child‐specific outcome measures have been developed to assess constructs such as ‘pain intensity’ via a face‐based scale, ‘pain behaviours’ and ‘functional disability’ relevant to particular age ranges, ‘concept of pain’ and ‘fear of pain’ via 5‐point Likert scales, and ‘pain duration’ via pain diaries. However, tools are neither available for all paediatric age groups nor for other pain‐related variables, such as localisation, pain quality, impact, context or meaning. Standardising the use of questionnaires with every child presenting with LBP is ideal, and numerous efforts around the world are aiming to do so. For example, in Australia and New Zealand, the Paediatric electronic Persistent Pain Outcomes Collaboration was established to benchmark pain clinics using a battery of commonly used questionnaires,^21^ and in the US, the Patient Reported Outcome Measurement Information System is used to assess a range of biopsychosocial constructs.^22^ An example of a large single‐centre database is the Pediatric‐Collaborative Health Outcomes Information Registry.^23^ For researchers, core domains and measures for clinical trials to treat pain in children and adolescents have been defined in the PedIMMPACT recommendations.^24^

For clinicians, it is important to critically evaluate the appropriateness of outcome measures for children and adolescents with LBP. The purpose for which a tool has been evaluated (e.g. discriminate between subgroups, predict outcomes and/or evaluate effectiveness) should be considered. In addition, the aspects of validity and/or reliability may have only been tested in a particular age range which may not generalise to a child being assessed in their clinic. Therefore, clinicians should weigh the clinical value and time burdens of consistently collecting questionnaire data, compared to occasionally collecting questionnaire data.

\Some clinics routinely commence assessments while a child is on a waiting list, by remotely administering patient‐reported outcome measures in advance of initial appointments. This strategy may improve the efficiency of an initial appointment. Also, clarifying questions can be asked regarding the data collected in the outcome measures to provide insights not otherwise easily collected. For example, if a child completes a pain diary prior to attending their initial appointment, they may report different symptoms to those discussed during the appointment. By clarifying these discrepancies, a better understanding of the pattern of symptoms is gained, increasing a clinician's confidence of ruling in or out a serious pathology.

### Treatment planning

The World Health Organization's International Classification of Functioning, Disability and Health provides useful categories for the problem list resulting from the assessment of children and adolescents with LBP. Table 3 outlines a brief example of a framework that could be used in creating a prioritised problem list to address with treatments. Iteratively creating this problem list may highlight that further assessments are required. For example, a standardised assessment of motor skills development or activities that are meaningful to the young person (e.g. COPM, GAS) may inform patient goals. Following guideline‐based physical, psychological and/or pharmacological treatments which have recently been meta‐analysed by the World Health Organization,^25^ a re‐assessment could be conducted to then plan future treatments.

**Table 3:** Developing a problem list out of assessment findings, mapping it to the International Classification of Functioning, Disability and Health, and considering specific physical treatment strategies

ICF category	Example problem list	Outcome measures	Categories of physical treatment strategies
Impairments	Pain, decreased AROM	Visual Analogue Scale, goniometry Medication use	Education, exercises, medication
Activity limitations	Limited walking, sitting and stairs endurance	Standardised time‐based measures, goal attainment scale	Part‐task practice, education, exercises
Participation restrictions	Unable to attend full day of school Taking part in meaningful activities	Short‐Form 36‐item questionnaire School attendance	Education re: Flare‐up planning, long‐term benefits of school/sport participation Liaison with school personnel or sport coach
Environmental factors	Bedroom upstairs and pain climbing stairs	Standardised time‐based measures, goal attainment scale	Part‐task practice, education, exercises
Personal factors	Tendency to enter ‘boom‐bust cycle’	Activity trackers, for example pedometer	Education re: Pacing. Exercises

AROM, active range of motion; ICF, International Classification of Functioning; re, regarding.

Short‐ and long‐term S.M.A.R.T. (specific, measurable, achievable, realistic and timely) goals can be collaboratively set based on the problem list, using motivational interviewing and shared decision‐making strategies. An example SMART goal with social, emotional and physical impact and considerations may be: ‘*In 4 weeks I aim to move between classes with everyone else at school so I am not leaving class early on my own, and climb the stairs rather than using the lift*’.

### *Re*‐assessment

Periodically throughout treatments, and prior to discharge from a health service, a systematic re‐assessment is essential to understand changes that have occurred and to consider whether any problems are beyond a clinician's scope of practice and require referral. By re‐assessing variables deemed clinically important from the initial assessment, the intention is that progress can be quantified. This provides valuable clinical feedback and aids in re‐prioritising the problem list and revising collaborative goals. Rigorous approaches to data collection and analysis to reduce bias in research, such as blinding assessments and re‐assessments, could also be attempted in clinical settings in the future.

## Conclusion

Systematically assessing children and adolescents seeking care for LBP is important and different to assessing an adult. Clinicians should consider the unique strategies required when assessing LBP in children and adolescents, such as identifying paediatric spinal pathologies, maximising a child's comfort while listening to their narrative and that of their guardians, the type and timing of child‐specific physical examination tests, and use of child‐specific outcome measures.
